# Biofabrication of pheochromocytoma and paraganglioma tumor organoids and assessment of response to systemic therapy

**DOI:** 10.1038/s41598-025-19806-w

**Published:** 2025-10-14

**Authors:** Richard A. Erali, Steven D. Forsythe, Cecilia R. Schaaf, Nicholas Edenhoffer, William Meeker, Cristian D. Valenzuela, Wencheng Li, Shay Soker, Reese W. Randle, Konstantinos I. Votanopoulos

**Affiliations:** 1 Wake Forest Organoid Research Center, Wake Forest University, Winston-Salem, NC USA; 2 Wake Forest Institute of Regenerative Medicine, Wake Forest University, Winston-Salem, NC USA; 3 Cancer Biology, Wake Forest University, Winston-Salem, NC USA; 4 Department of Comparative Medicine, Wake Forest University School of Medicine, Winston- Salem, NC USA; 5 Department of Pathology, Atrium Health Wake Forest Baptist, Winston-Salem, NC USA; 6 Division of Surgical Oncology, Department of Surgery, Atrium Health Wake Forest Baptist, Winston-Salem, NC USA; 7 Wake Forest Department of General Surgery, 1 Medical Center Blvd, Winston-Salem, NC 27157 USA

**Keywords:** Pheochromocytoma, Paraganglioma, Tumor organoids, Organoids, Precision oncology, Cancer models, Adrenal gland diseases, Surgical oncology, Chemotherapy, Molecularly targeted therapy

## Abstract

Introduction Pheochromocytoma (PCC) and paraganglioma (PG) research is limited due to rarity of disease. We utilized patient-derived tumor organoids (PTOs) to explore personalized treatment options for these patients. Tumors were obtained from patients with PCC and PG who underwent operative resection and processed to biofabricate PTOs utilizing a collagen-based hydrogel. Blood was also obtained from patients to generate immune-enhanced PTOs (iPTOs). Organoids underwent treatment with chemotherapy, immunotherapy, or tyrosine kinase inhibitors. Cell viability was determined using multiple assays. Significant treatments demonstrated viability < 50% and p < 0.05 compared to controls. Twelve PCC and 4 PG tumors were obtained from December 2020 – May 2023. Viable PTOs were generated in 15/16 (93.8%) tumors for drug studies. Sunitinib (10 µM) demonstrated significant treatment efficacy in 9/14 PTOs (64.3%) with average post-treatment viability of 28%. A commonly utilized clinical regimen of cyclophosphamide, vincristine, doxorubicin and dacarbazine (10 µM) demonstrated significant treatment effect in 3/7 treated PTOs (42.9%) with average post-treatment viability of 25.5%. Only 1/13 iPTOs (7.7%) demonstrated sensitivity to Ipilimumab/Nivolumab. Cultured PTOs produced measurable norepinephrine on ELISA. PTOs are a feasible platform to study PCC and PG and their response to systemic treatments on a personalized level.

**Supplementary Information:**

The online version contains supplementary material available at 10.1038/s41598-025-19806-w.

## Introduction

 Pheochromocytomas (PCCs) and paragangliomas (PGs) are rare neuroendocrine tumors arising from the adrenal medulla and autonomic nervous system paraganglia, respectively. Together, there are an estimated 0.8 cases per 100,000 persons and about 10% of PCCs and 25% of PGs demonstrate metastasis on presentation^1,2^. Patients with isolated metastases to the liver or bone may be treated with locoregional options such as radiation therapy, ablation, or transarterial chemoembolization (liver only)^3–5^. Surgery is the only curative treatment option and there are limited systemic therapies for patients with metastatic disease^6,7^. Newer systemic therapy options are on the horizon, including the newly FDA-approved belzutifan for metastatic PCC/PG, but has not been studied extensively^8,9^. Iobenguane 131- metaiodobenzylguanidine (I-131 MIBG) was first utilized for PCC/PG treatment in 1983 as a low specific activity format but never gained FDA approval^10^. High specific activity I-131 MIBG has been increasingly studied since its FDA approval in 2018 but is not widely available^10–13^.

Given the rarity of this tumor, there is a paucity of clinical data regarding the efficacy of systemic treatment options. To date, the largest study comprises 52 patients over a 31-year period with an objective response in 17/52 (33%) patients with responders experiencing improved survival (6.4 vs. 3.7 years) with combinations of cyclophosphamide, vincristine, dacarbazine and doxorubicin^6^. Although no prospective clinical trials have been performed with this regimen, more recent studies have supported its continued use^14,15^. Sunitinib and Cabozantinib are tyrosine kinase inhibitors of the VEGF receptor and have demonstrated improved survival and tumor response rates (measured by RECIST criteria)^16–19^. The FIRSTMAPPP trial demonstrated improved progression free survival with Sunitinib compared to placebo, highlighting another treatment option for patients with metastatic disease.

Three-dimensional (3D) culture methods have become increasingly utilized to study cancer due to their ability to mimic tissue histology, maintain tumor heterogeneity, and accurately recapitulate the tumor microenvironment^20^. Amongst these, 3D spheroids and tumor organoids are commonly employed. Both of these culture methods can utilize patient-derived tumor tissue; however, spheroid culture, while faster forming, is typically used for rapid testing and not considered for long term culture, while ECM supported organoids can be propagated for long term experimentation^20,21^. PTOs can be utilized as a screening tool to generate individualized treatments based on PTO drug sensitivity. In this way, PTOs biofabricated from a single patient can generate multi-drug panel sensitivities rather than fragment study populations into randomized clinical trials examining the effects of a few drugs. Thus, PTO studies could accelerate clinically meaningful research progress beyond what is currently possible^22^.

Herein, we present, to our knowledge, one of the first manuscripts outlining the biofabrication, culture, and drug screening of PCC and PG patient-derived tumor organoids—generating data within 10 days of tissue acquisition. In addition to viability changes, we also assessed the biochemical response to treatments.

## Methods

### Patient tumor & whole blood specimens

Tissue and whole blood specimens were obtained from 12 patients with PCC and 4 with PG who underwent surgical resection at our institution between December 2020 and May 2023. Specimens were obtained in accordance with Atrium Health Wake Forest Baptist Medical Center guidelines and under an established institution-approved IRB protocol. Informed consent was obtained from all patients in this study. Resected tissue specimens were placed in Roswell Park Memorial Institute (RPMI) media and transferred to the Wake Forest Organoid Research Center (WFORCE) for processing within 2 h from surgical resection.

## Tumor & whole blood processing

Our tumor processing protocol has been previously published in detail^23–25^. Briefly, on arrival to the laboratory, tumors were washed for two 5-minute cycles in an antibiotic and anti-mycotic solution. After washing, a thin cross-section of each specimen was obtained for whole tissue histology. The remaining tissue specimen was minced finely and placed in a digestive enzymatic solution containing Dulbecco’s Modified Eagle’s Medium (DMEM) with 100,000 cytidine deaminase (CDA) units per mL collagenase HA (001-1050; VitaCyte, Indianapolis, IN), 22,000 narcissus pseudonarcissus agglutinin (NPA) units per mL protease (003-1000; VitaCyte, Indianapolis, IN) for up to 120 min under agitation at 37 °C. Upon termination of enzymatic digestion, the tumor solution was filtered and then centrifuged to isolate a tumor cell pellet. Red blood cell lysis was performed on the pellet and the resultant cells were counted using a NucleoCounter NC-200 (Chemometec, Denmark). Immunocompetent cells were isolated from each corresponding patient’s whole blood using Ficoll-Paque PLUS according to company procotol (GE Healthcare, Chicago, US).

## Organoid fabrication & culture

The tumor cell pellet was resuspended with a hydrogel solution containing thiol-modified hyaluronan/heparin (Heprasil^®^; Advanced Biomatrix, San Diego, CA) and methacrylated collagen (PhotoCol^®^; Advanced Biomatrix, San Diego, CA) in a 1:3 volume ratio at a cell density of 10 million cells per mL. Organoids were biofabricated by seeding 5 µL of the hydrogel/cell suspension into individual wells of a non-tissue culture treated 96-well plate and then exposed to UV light (365 nm, 18 W/cm^2^) from a BlueWave 75 V.2 UV spot lamp (Dymax Corp., Torrington, CT) for 1 s to crosslink the hydrogel. PTOs were cultured in 200 µL DMEM-F12 based organoid media previously reported^23^.

Immune-enhanced PTOs (iPTOs) were created by combining peripheral blood mononuclear cells (PBMCs) from each patient’s corresponding whole blood in a ratio of 1:5 (Tumor: PBMCs) prior to resuspension with the hydrogel solution. Biofabrication and culture methods were similar to PTOs. Organoids were cultured for 7 days prior to treatment.

## Chemotherapy, tyrosine kinase inhibitors & immunotherapy drug screens

After 7 days of culture, PTOs were treated with several clinically utilized chemotherapy agents^6,7,26,27^. The following cytotoxic drug combinations and doses were used: (1) Vincristine (V8879, Sigma-Aldrich), Dacarbazine (S1221 SelleckChem), Doxorubicin (S1208, SelleckChem) (0.1/0.1/0.1 µM, 1/1/1 µM, and 10/10/10 µM), (2) Vincristine, Dacarbazine, Doxorubicin, Cyclophosphamide (19527, Cayman Chemical) (0.1/0.1/0.1/0.1 µM, 1/1/1/1 µM, and 10/10/10/10 µM), (3) Gemcitabine (G6423, Sigma-Aldrich), Paclitaxel (T7402, Sigma-Aldrich) (10/10 µM, 50/50, µM 100/100 µM), and (4) Temozolomide (T2577, Sigma-Aldrich) (10 µM, 100 µM, 1000 µM). Organoids were also treated with a variety of receptor tyrosine kinase inhibitors including Sunitinib (S1042, SelleckChem), Pazopanib (S3012, SelleckChem), Cabozantinib (S4001, SelleckChem)^16^ in logarithmic dosing intervals at 0.1 µM, 1 µM and 10 µM. Sunitinib pharmacokinetic studies evaluating therapeutic efficacy have identified drug plasma *trough* concentrations ranging from 50 ng/mL to 100 ng/mL^28,29^, which correlates to a molarity of 0.125µM − 0.250µM, respectively. These trough dose ranges were captured by our 3-tier logarithmic dosing of 0.1µM, 1µM and 10µM.

Similarly, PTOs were treated with immunotherapy agents: 100 nM of Pembrolizumab (A2002, Selleckchem, Houston, TX), Ipilimumab (A2001, Selleckchem, Houston, TX) or Nivolumab (A2005, Selleckchem, Houston, TX).

Prior to treatment, spent media was aspirated from the wells and replaced with drug solutions mixed in culture media. Organoids remained in drug-containing media solution for 72 h prior to endpoint viability assessment.

## Organoid viability assessment

Similar to previously published protocols, PTO’s viability was assessed with LIVE/DEAD staining and ATP viability assays^30^. Briefly, LIVE/DEAD staining was performed (L3224; Invitrogen, Carlsbad, CA) according to the manufacture’s protocol and whole-organoid imaging performed with a Leica TCS LSI macro confocal microscope (Leica Microsystems Inc., Buffalo Grove, IL). Final images represent both the red and green channels overlaid and stacked in maximum projection.

Additionally, we performed quantitative viability with the CellTiter-Glo^®^ 3D Cell Viability Assay (G968B; Promega, Madison, WI) as previously described^30^. Measurements were conducted in a Costar White Polystyrene 96 well Assay Plate (3912; Corning, NY) and analyzed with a Veritas Microplate Luminometer (Turner BioSystems, Sunnyvale, CA).

### Definition & assessment of treatment response

When defining treatment response, we describe the response based on “post-treatment viability” which refers to the percentage of cells alive after treatment. For example, a post-treatment viability of 90% would mean only 10% of cells were killed with treatment. “Significant responses” for non-immunotherapy treated PTOs were defined by significantly less cell viability (*p* < 0.05 and < 50% post-treatment viability) compared to non-treated controls.

Based on our previous iPTO experience^30^we defined successful immunotherapy efficacy (i.e. cell killing) when iPTOs met the following criteria: (1) exhibited a post immunotherapy ATP viability < 50%, and (2) significantly less cell viability (i.e. *p* < 0.05) compared to both the immune matched controls (Ex: iPTO control vs. iPTO treated) and non-immune enhanced counter condition (Ex: Pembrolizumab treated iPTO vs. Pembrolizumab treated PTO).

## Assessment of biochemical response

Systemic therapy for PCC and PG can help control symptoms from excess catecholamines, which may be beneficial for patients with unresectable tumors or high tumor burden^6,16^. For Patient 10, we analyzed catecholamine levels in the PTO media after treatment with tyrosine kinase inhibitors using an epinephrine/norepinephrine ELISA kit (KA1877, ABNOVA). On day 10 (termination of drug screen study), we aspirated media from treated and untreated wells for testing which was conducted according to manufacturer protocol. Absorbance was measured with a Veritas Microplate Luminometer (Turner BioSystems, Sunnyvale, CA) and post-treatment values were interpolated from a standard curve generated with GraphPad Prism (GraphPad Software Inc., USA).

## Organoid tissue characterization

Organoids were fixed for histology on culture days 1 and 10 for 4 h in 4% paraformaldehyde. Organoids were paraffin embedded, sectioned at 5- µm intervals, and stained on glass microscope slides with hematoxylin and eosin (H&E).

Additional staining was performed with immunohistochemistry (IHC) to characterize Chromogranin A (1:250, ab15160, Abcam, rabbit), S100 (1:200, ab183979, Abcam, rabbit), and synaptophysin (1:200, MA5-14532, Invitrogen, rabbit) biomarker expression according to previously published protocols^30^. Sections were imaged with an Olympus BX-63 upright fluorescent microscope (Olympus, Tokyo, Japan).

### Statistical analysis

All data are expressed as mean ± standard deviation for each experimental group. Analyzed treatment groups consisted of 3 or more organoids. Prior to statistical analysis, ATP assay values were standardized to a control value of 100 for ease of interpretation. To compare the ATP viability between independent treatment groups, we performed one-way ANOVA with Šídák’s test to correct for multiple comparisons. We deemed a drug screen successful based on adequate day 10 viability of the treatment and control conditions. GraphPad Prism (GraphPad Software Inc., USA) was used to conduct statistical analysis with a p value of < 0.05 as the threshold for statistical significance. Grubbs’ method was utilized within GraphPad to identify outlier values with an Alpha = 0.1.

## Results

### Patient & tumor characteristics

**Fig. 1:**
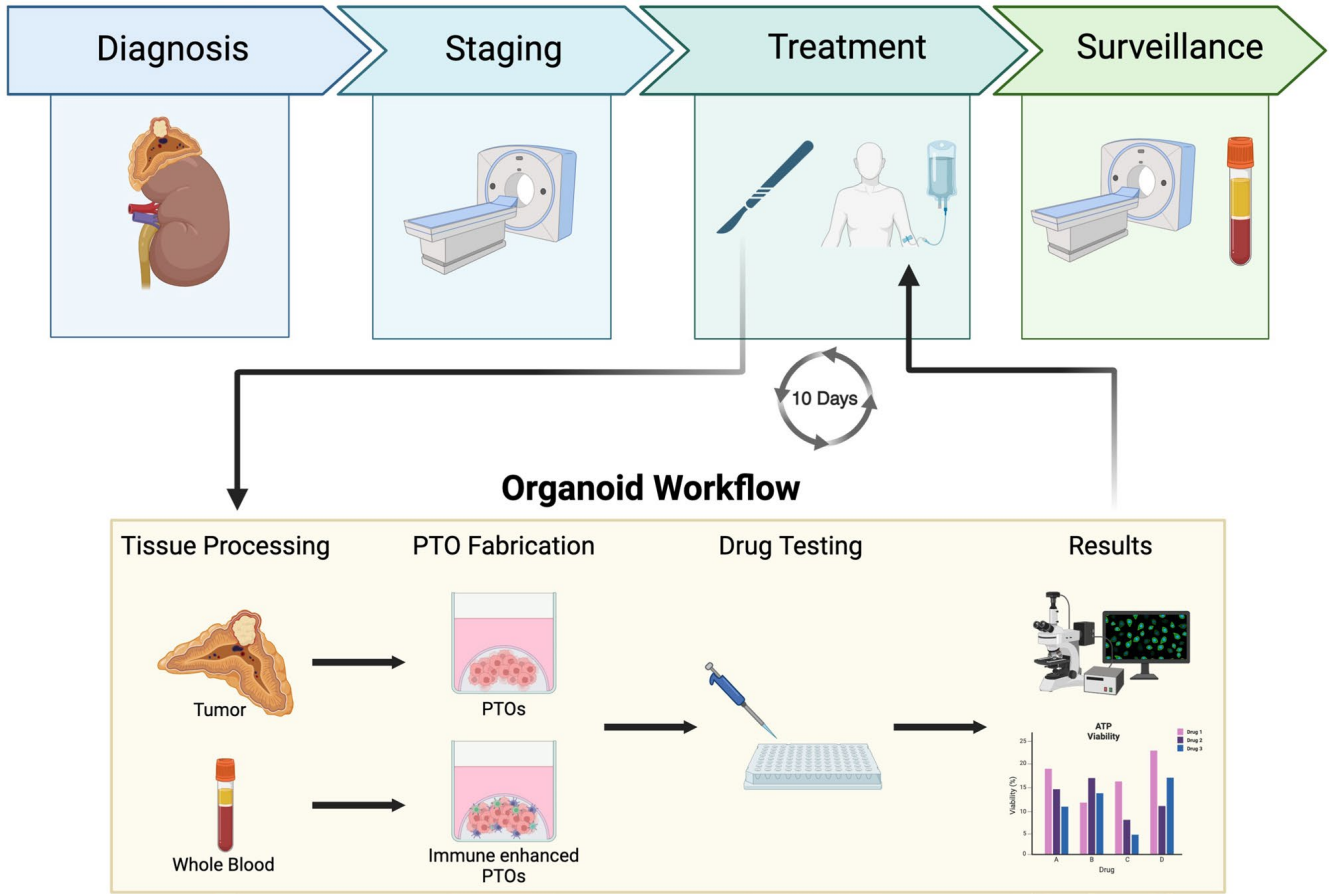
Workflow diagram of tissue processing and organoid biofabrication process. Created with BioRender.com.

A total of 16 tumors from 15 patients were utilized in this study from December 2020 to May 2023 (Table 1). Four patient’s tumors were PGs (25%) and 12 were PCCs (75%). Tumors were transferred to Wake Forest Organoid Research Center (WFORCE) within 2 h of resection. Overall, results generated within 10 days of surgery (Fig. 1). Patient 1 presented with metastatic PCC and underwent resection of the primary tumor in attempt to mitigate the risk of excess catecholamines. Patient 3 developed recurrence after their initial operation and underwent repeat resection which we obtained for comparison to the original tumor (Tumor 3 − 2). No patients in the cohort received neoadjuvant systemic therapy. Patient demographics including race/ethnicity are not reported due to the possibility of patient identification in this rare disease cohort.

**Table 1:** Patient and tumor clinical information along with PTO drug screens utilized for each. Tumors are pheochromocytomas except where indicated (PG – paraganglioma). FoundationOne and genetic testing was performed at the discretion of the treating clinician. SDH – Succinate dehydrogenase, VUS – variant of uncertain significance, BLM – Bloom syndrome, NF – Neurofibromatosis, VHL – Von Hippel lindau, RT – Radiation therapy, NED – No evidence of disease, N/A – Not applicable, PASS - The pheochromocytoma of the adrenal gland scaled score. DOD – died of disease. NED – no evidence of disease. Gem/Pac – Gemcitabine and paclitaxel. Tem – Temozolomide. VDD – Vincristine, dacarbazine, doxorubicin, VDDC – Vincristine, dacarbazine, doxorubicin, cyclophosphamide. TKIs – Tyrosine kinase inhibitors.

Tumor (Type)	FoundationOne, Genetics Testing	Prior Treatment	PASS	Tumor Size (cm)	Current Clinical	PTO Drug Screen
**1**	SDH-B	RT (neck, hip)	10	16.8	DOD	Gem/Pac, Tem
**2**	N/A		3	6.4	NED	Gem/Pac, Tem, Immunotherapy
**3 − 1 (PG)**	VUS (BLM gene)			9	Recurrence, Re-resection (Tumor 3 − 2). Clinical trial enrollment pending	TKIs, Immunotherapy
**3 − 2 (PG)**		Surgery		9.6		VDDC, TKIs, Tem
**4**	Negative		10	15	NED	Gem/Pac, VDD, Tem, TKIs, Immunotherapy
**5 (PG)**	SDH-A (Invitae)			6	NED	Gem/Pac, VDD, Tem, TKIs, Immunotherapy
**6**	N/A		3	3	NED	VDD, TKIs, Immunotherapy
**7**	SDH-B		7	10	Recurrence, on Axitinib	Gem/Pac, VDD, Tem, TKIs, Immunotherapy
**8**	VHL-1		2	7.5	NED	VDD, Tem, TKIs, Immunotherapy
**9**	Negative		9	6.4	NED	Gem/Pac, VDD, TKIs, Immunotherapy
**10 (PG)**	NF-1			6	NED from PG. Diagnosed with Stage IV ovarian cancer	VDDC, TKIs, Immunotherapy
**11**	RAD50		10	4.2	NED	VDDC, Tem, TKIs, Immunotherapy
**12**	N/A		6	4.4	NED	Gem/Pac, VDDC, TKIs, Immunotherapy
**13**	Negative		5	3.4	NED	VDDC, TKIs, Immunotherapy
**14**	NF-1		1	3.5	NED	VDDC, TKIs, Immunotherapy
**15**	Negative		2	3.5	NED	VDDC, TKIs, Immunotherapy

Genetic testing was conducted according to clinical guidelines after a consultation with a genetic counselor and consent was obtained. The Invitae Hereditary Paraganglioma-Pheochromocytoma Panel, Invitae Multi-Cancer Panel, Invitae Common Hereditary Cancers Panel, and FoundationOne Dx were most utilized tests (Supplemental Table 1). Three patients (18.8%) did not undergo genetic testing, 4 patients had negative results on gene panel testing (25%), 1 patient had SDH-A pathogenic variant (PV) (6.3%), 2 patients had SDH-B (PV) (12.5%), 2 patients with NF-1 (PV) (12.5%), 1 with VHL (PV) (6.3%), 1 with RAD50 (PV) (6.3%), and 1 patient with variance of uncertain significance (BLM, 6.3%). Currently, one patient died of disease (6.7%), two experienced recurrence (13.3%), and 12 have no evidence of disease (80%) at last clinic follow-up (Table 1).

**Fig. 2:**
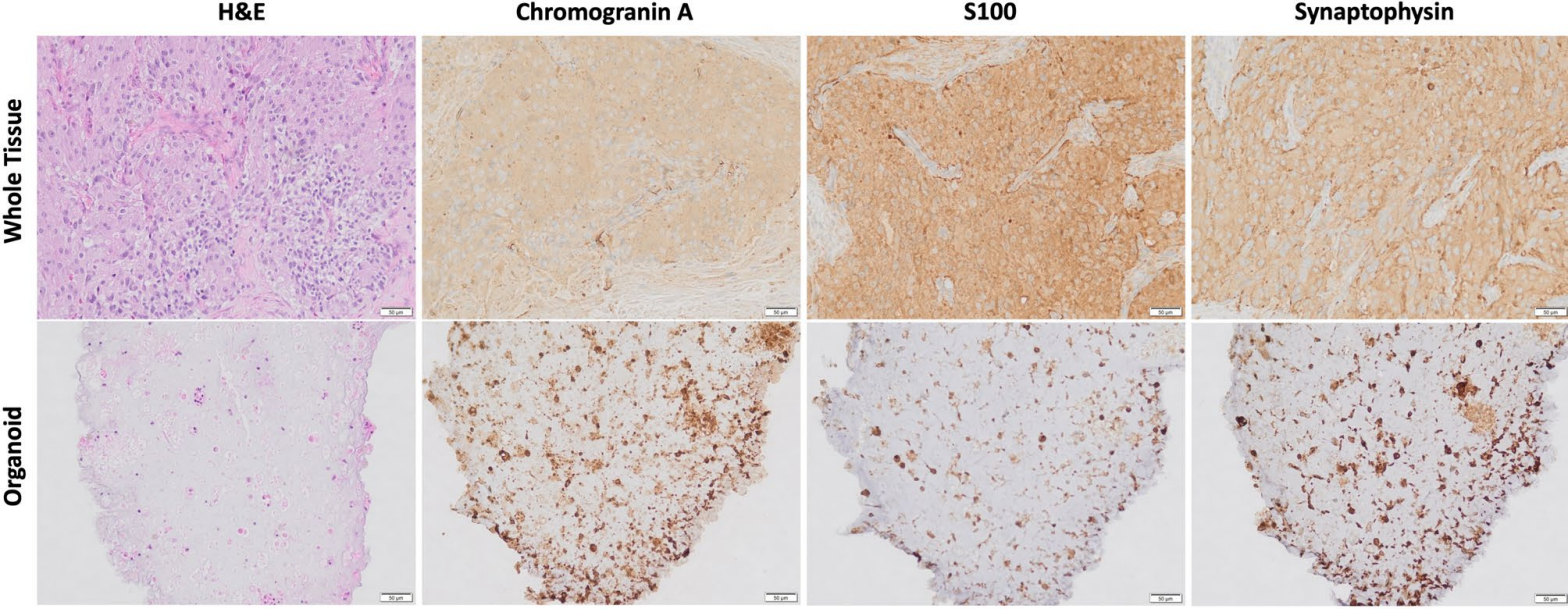
Comparative histological staining of whole tissue and organoids from Patient 3. Hematoxylin and eosin, Chromogranin A, S100, and Synaptophysin shown at 20X magnification. Organoids sectioned after 10 days in culture.

Tumor organoids demonstrated high histological fidelity with native tumor tissue when stained with hematoxylin and eosin, revealing nests of tumor cells with basophilic to clear cytoplasm and mild nuclear pleomorphism. More importantly, both the organoids and native tissue showed positivity for PCC/PG markers such as Chromogranin A, S100, and Synaptophysin by immunohistochemistry (Fig. 2). Chromogenic staining for Chromogranin A and Synaptophysin was performed on tumors 1–10. S100 staining was performed on tumors 1–9.

### Chemotherapy response

**Fig. 3:**
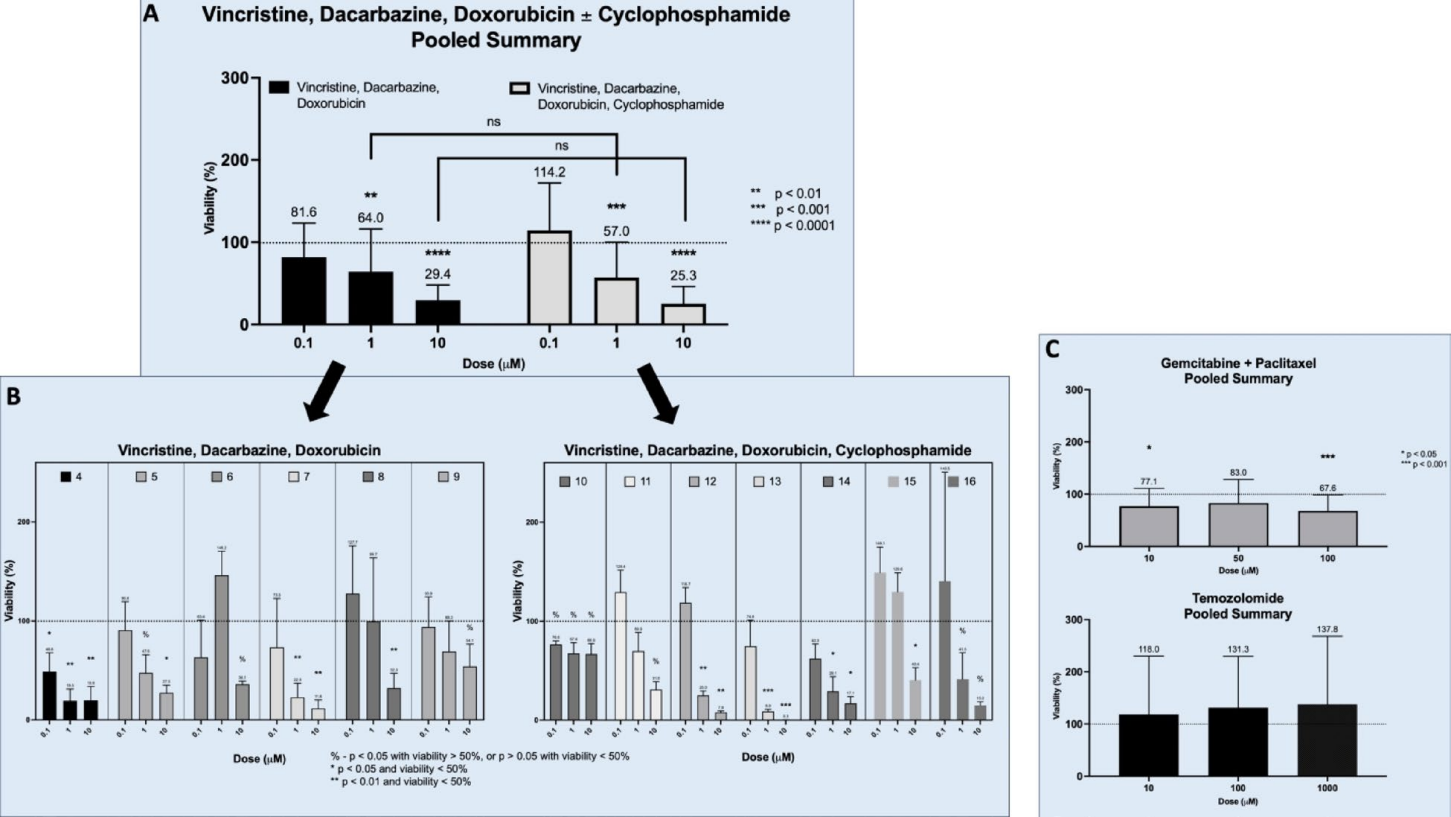
**(A)** Pooled summary ATP viability data of PTOs treated with Vincristine, Dacarbazine, Doxorubicin (VDD) and Vincristine, Dacarbazine, Doxorubicin, and Cyclophosphamide (VDDC). *N* = 5 per patient’s PTO. Patients 4–16 replicates were pooled for summary data. There was no statistically significant improved cytotoxicity with the addition of cyclophosphamide at 1 and 10 μm doses. **(B)** Individual PTO viability results for patients treated with VDD (Patients 4–9) and VDDC (patients 10–16). PTOs were treated in replicates of *n* = 5. **(C)** Pooled summary ATP viability data of PTOs treated with Gemcitabine + Paclitaxel, and Temozolomide. Each patient’s PTO treated with Gemcitabine/Paclitaxel and Temozolomide were treated in replicates of *N* = 5 and pooled for summary graph shown. Average viability shown above each treatment dose with error bars representing standard deviation. Y-axis is viability as a percent of the control for each patient’s PTOs. X-axis is the dose (µM). The horizontal dotted line represents untreated control viability of 100%. p-values defined by each representative chart.

**Fig. 4:**
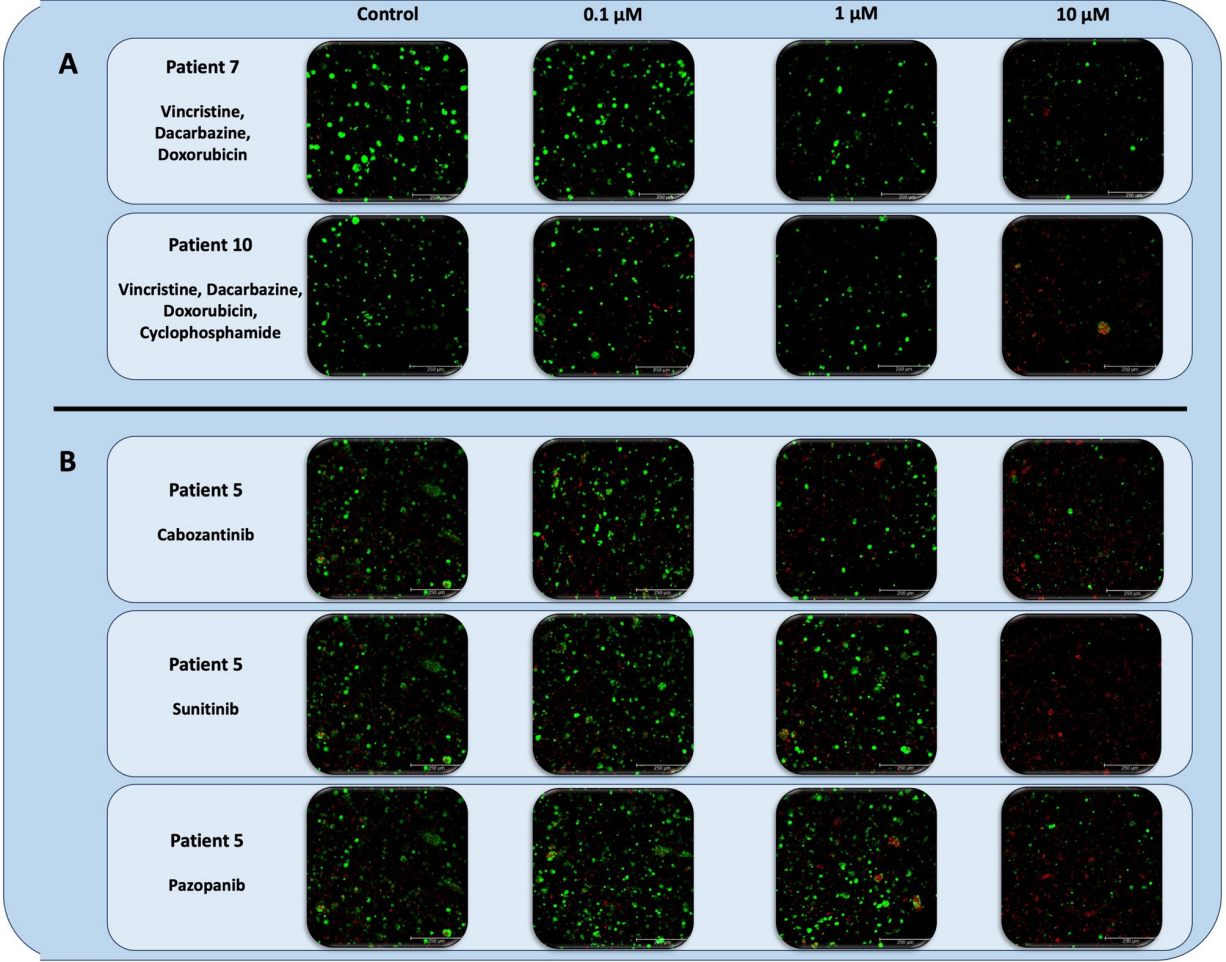
Live dead imaging of: **A)** Patient 7 and 10 organoids after treatment with the combination Vincristine, Dacarbazine, Doxorubicin ± Cyclophosphamide. **B)** Patient 5 organoids after treatment with tyrosine kinase inhibitors: Cabozantinib (top row), Sunitinib (middle row), and Pazopanib (bottom row). Untreated control image served as control for all three drug treatments during experiment. Doses indicated at the top of each column. Live cells green; dead cells red. Scale bar 250 μm.

Dacarbazine-based chemotherapy is recommended for the treatment of metastatic PCC and PG, based on long-term institutional data published by MD Anderson^6^. We utilized two regimens in our cohort: Vincristine, Dacarbazine, Doxorubicin (VDD) and Vincristine, Dacarbazine, Doxorubicin, and Cyclophosphamide (VDDC) (Fig. 3A). Due to COVID-19 drug shortages, we were unable to include Cyclophosphamide for patients 4–9. On pooled analysis, VDD demonstrated significant treatment responses at 1 µM and 10 µM doses with post-treatment viabilities of 64% (*p* < 0.01) and 29.4% (*p* < 0.0001), respectively. Similarly, VDDC demonstrated significant treatment responses at 1 µM and 10 µM doses with post-treatment viabilities of 57% (*p* < 0.001) and 25.3% (*p* < 0.0001), respectively. There was no statistical difference between VDD and VDDC post-treatment viabilities at 1 µM and 10 µM doses (Fig. 3A). Overall, at the individual patient level, tumors tested with VDD demonstrated significant responses in 4/6 instances (66.7%) and in 4/7 (57.1%) in those treated with VDDC (Fig. 3B). Live dead imaging is shown for Patients 7 (VDD) and 10 (VDDC) demonstrating the qualitative cytotoxicity of each regimen (Fig. 4A).

We also conducted drug screens with Gemcitabine and Paclitaxel^26,27^. Gemcitabine and Paclitaxel demonstrated less cytotoxic activity compared to VDD and VDDC, based on post-treatment viability. On pooled analysis, Gemcitabine and Paclitaxel treatment resulted in post-treatment viability of 77.1% at 10 µM (*p* < 0.05), 83% (ns), and 67.6% at 100 µM (*p* < 0.001) (Fig. 3C).

Temozolomide has been previously reported to demonstrate response in patients with succinate dehydrogenase B (SDH-B) PVs^31^. We did not identify post-treatment responses to Temozolomide on pooled analysis (Fig. 3C). In Patients 1 and 7, both with SDH-B PVs, the average post-treatment viability was 100.1% (ns) and 72.7% (ns) at 1000 µM, respectively.

### Tyrosine kinase inhibitor response

**Fig. 5:**
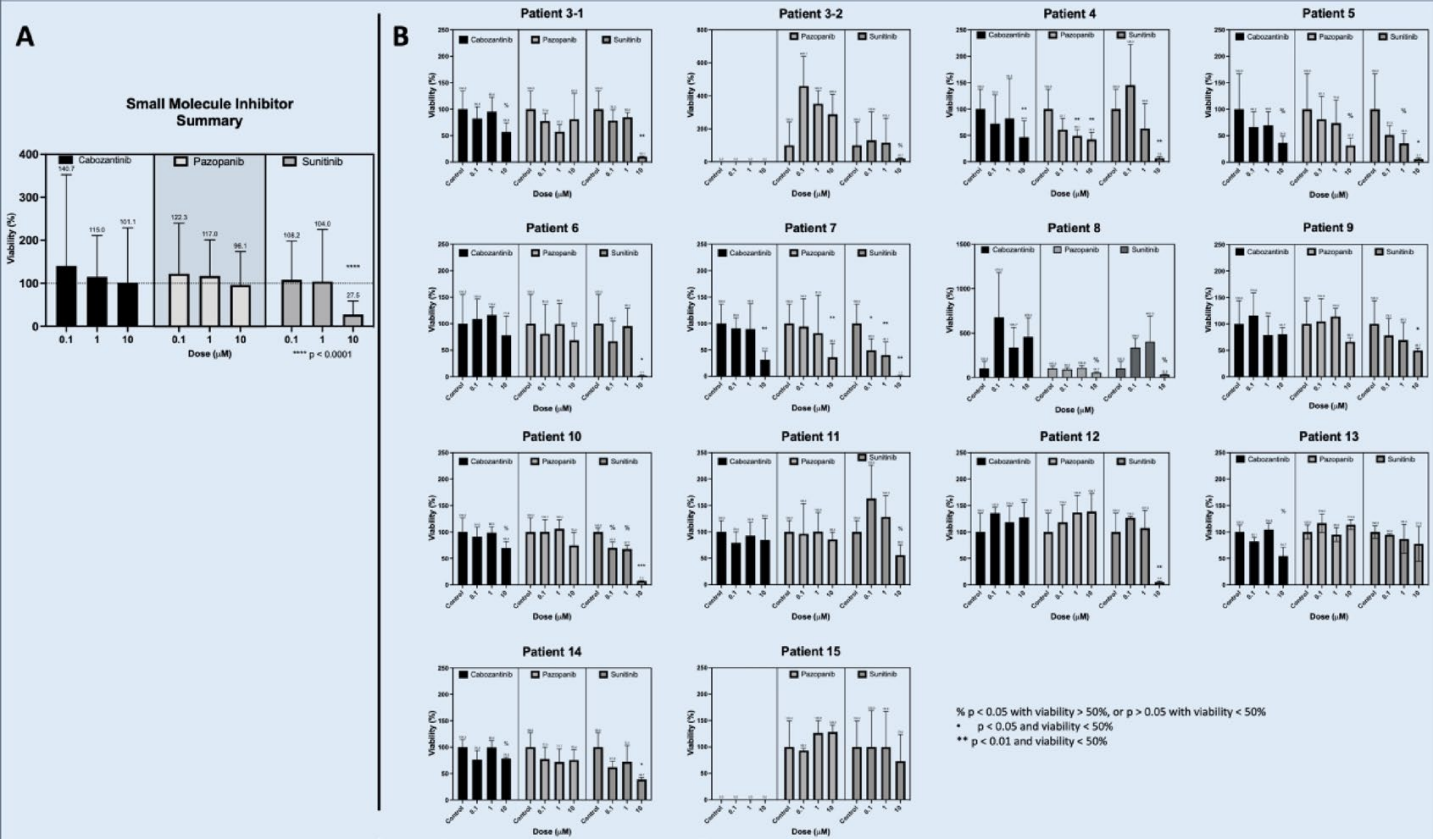
**(A)** Pooled summary ATP viability data of PTOs treated with tyrosine kinase inhibitors Cabozantinib, Pazopanib, and Sunitinib. Pooled average viability shown above each treatment dose for all PTOs treated with drug. The horizontal dotted line represents untreated control viability of 100%. Each patient’s PTO replicates were pooled for analysis in the summary graph shown. **(B)** Individual PTO viability data from Patients 3–15 treated with tyrosine kinase inhibitors Cabozantinib, Pazopanib, and Sunitinib. Patients 3 − 2 and 15 PTO’s were not treated with Cabozantinib. Each patient’s PTOs were treated in replicates of *n* = 5. Average viability shown above each treatment dose. Error bars represent standard deviation. Y-axis is viability as a percent of the control for each Patient’s PTOs. X-axis is the dose (µM), controls were untreated.

There has been growing evidence to support the use of Sunitinib in the treatment of patients with metastatic PCC and PG^16–18^. In this study, we examined the efficacy of Sunitinib in 14/16 tumors and identified a significant response in 9/14 (64.3%) with an average post-treatment viability of 27.5% at 10 µM (*p* < 0.0001) (Fig. 5). We also tested efficacy of similar small molecule inhibitors Cabozantinib and Pazopanib. Cabozantinib demonstrated response in 2/12 (16.7%) with an average post-treatment viability of 101.1% at 10 µM (ns). Pazopanib demonstrated response in 2/14 (14.3%) with an average post-treatment viability of 96.1% at 10 µM (ns) (Fig. 5). Live dead imaging was performed for Cabozantinib, Pazopanib and Sunitinib, demonstrating the qualitative cytotoxicity of these agents in Patient 5’s PTOs (Fig. 4B).

### Immunotherapy response

We also tested Pembrolizumab and Nivolumab (PD-1 inhibitors) along with Ipilimumab (CTLA-4 inhibitor) in PCC and PG PTOs. Overall, 1/13 (7.7%) patients demonstrated significant treatment effect. Only Patient 5’s PTO treatment with Ipilimumab/Nivolumab resulted in significant response with post-treatment viability of 10% (Supplemental Fig. 1).

### Response stratified by patient & tumor characteristics

**Fig. 6:**
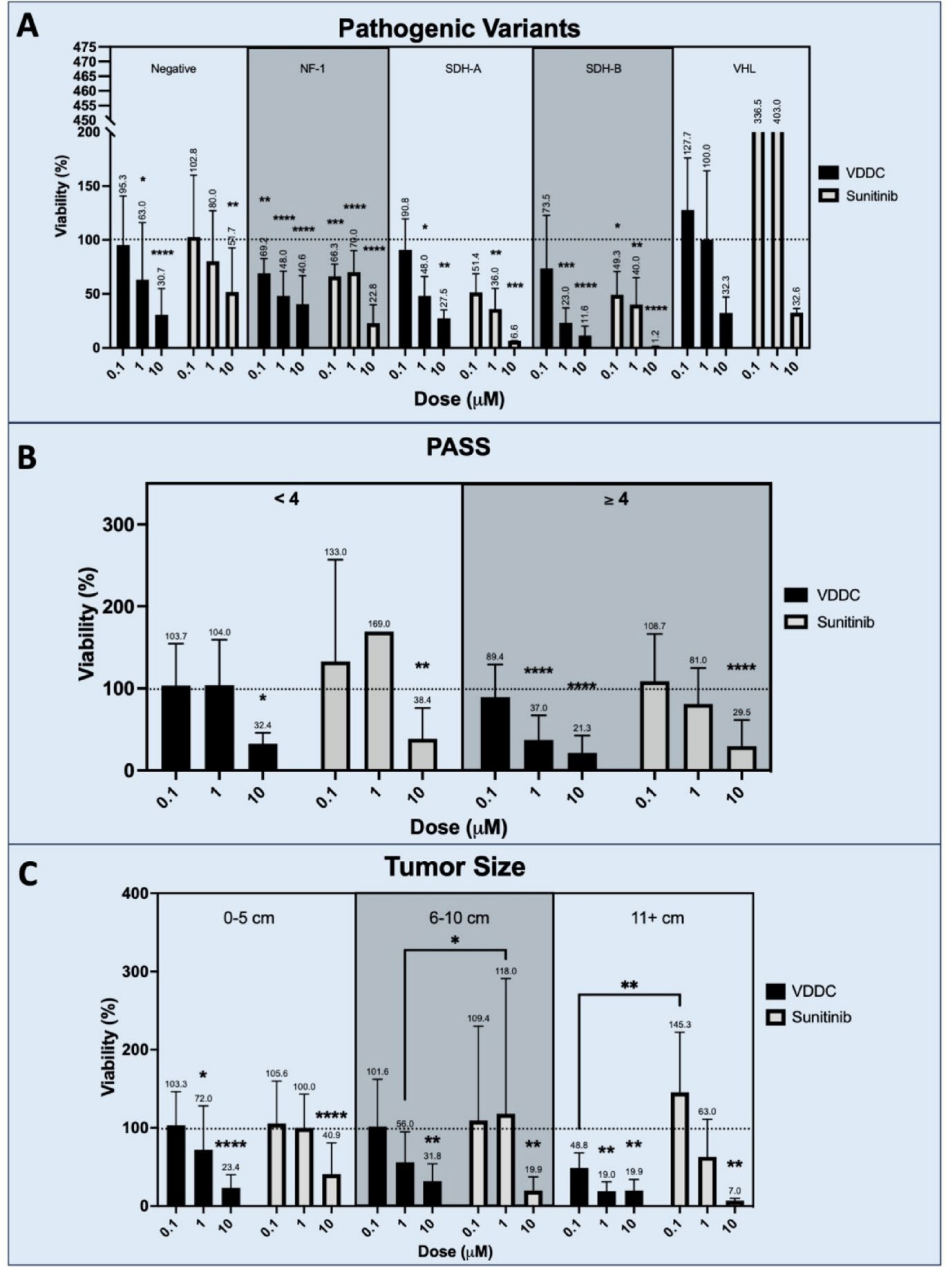
**A)** Comparison of PTOs treated with Vincristine, Dacarbazine, Doxorubicin ± Cyclophosphamide (VDD or VDDC) or Sunitinib sorted by mutation/syndrome status noted in Table 1. VHL 1 μm dose standard deviation bar exceeded Y-axis—this tumor demonstrated high variability amongst replicates. **B)** Comparison of Vincristine, Dacarbazine, Doxorubicin ± Cyclophosphamide (VDDC) and Sunitinib post-treatment PTO viability by PASS score (The Pheochromocytoma of the Adrenal Gland Scaled Score). **C)** Comparison of Vincristine, Dacarbazine, Doxorubicin, and Cyclophosphamide (VDDC) and Sunitinib post-treatment viability by tumor size. For each graph, Y-axis is viability as a percent of the control for each Patient’s PTOs. X-axis is the dose (µM). The horizontal dotted line represents untreated control viability of 100%. Statistical significance indicated above each dose is in comparison to untreated control. * *p* < 0.05, ** *p* < 0.01, *** *p* < 0.005, **** *p* < 0.0001.

Several patient and tumor characteristics have been demonstrated to inform patient’s prognosis including germline pathogenic variants/syndromes, pheochromocytoma of adrenal gland scaled score (PASS), and tumor size^32,33^. We compared the efficacy of VDDC and Sunitinib within each category. Subgroup analysis of PVs and syndromes revealed similar efficacy of VDDC and Sunitinib against each of the PVs in our cohort (NF-1, SDH-A, SDH-B, and VHL) (Fig. 6A). There was improved post-treatment response for SDH-A and SDH-B tumors with Sunitinib. When stratifying the response by PASS score, we found treatment with VDDC resulted in lower post-treatment viability compared to Sunitinib at 1 µM dose (*p* < 0.05) for PASS ≥ 4 (Fig. 6B). Finally, VDDC demonstrated lower post-treatment viabilities at 1 µM compared to Sunitinib for tumors 6–10 cm (*p* < 0.05) and at 0.1 µM for tumors 11 + cm (*p* < 0.01) (Fig. 6C).

### Biochemical response

**Fig. 7:**
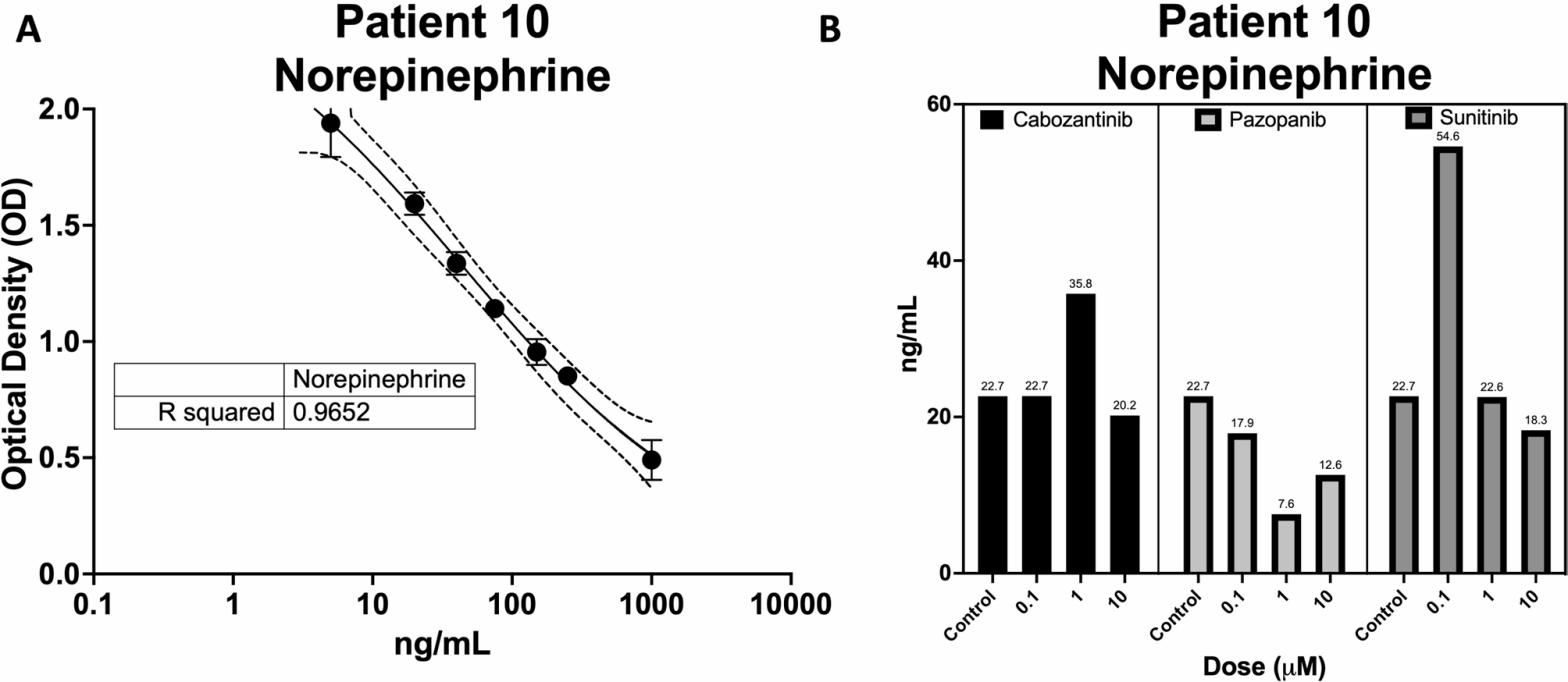
**(A)** Standard curve generated from Norepinephrine (NE) ELISA kit. **(B)** Patient 10 PTO Norepinephrine concentrations after treatment with Cabozantinib, Pazopanib, and Sunitinib. Untreated controls were also measured for NE levels. Values shown are calculated interpolated mean values of treated replicates from the standard curve shown in panel A. Y-axis is NE concentration in ng/mL. X-axis is dose in µM.

We analyzed the biochemical response in Patient 10’s PTO to small molecule inhibitors by measuring norepinephrine levels on day 10 after treatment (Fig. 7). Patient 10 demonstrated elevated levels of plasma metanephrines and normetanephrines pre-operatively. We found the PTOs produced small amounts of norepinephrine ex-vivo, which decreased with increasing doses of Cabozantinib, Pazopanib, and Sunitinib (Fig. 7).

## Discussion

The rarity of PCC and PG has made it difficult to generate level 1 evidence for new treatment advances^22,23,30,34–36^. Low incidence of disease can be overcome by incorporation of these rare tumors in prospective PTO studies—generating drug efficacy data to help inform treatment decisions, improve enrollment in clinical trials for those tumors resistant to standard of care, and investigate new drug applications both at the cohort and individual patient levels. In this study, we report the successful biofabrication of PCC and PG PTOs and present a clinically feasible workflow outlining the utility of PCC and PG PTOs to study responses to systemic treatments.

We have demonstrated the feasibility of utilizing patient-derived tumor organoids (PTOs) to study treatment efficacy in multiple cancer types including appendiceal cancer, colorectal cancer, Merkel cell carcinoma, peritoneal mesothelioma, and sarcomas^23–25,30,35–38^. Two previous groups have explored treatment responses of cultured pheochromocytoma tissue^39,40^. Bornstein et al. generated patient-derived spheroids from a single pheochromocytoma tumor and treated with gemcitabine^39^. Their work centered mostly on exploring the utility of a novel spheroid culture plate and did not quantify treatment response to the single drug studied. Wang et al. also successfully cultured human pheochromocytomas and paragangliomas as spheroid cultures for rapid testing, although these cultures were not validated using immunohistochemistry, genomics, or biochemical methods^40^. Our study is the first to validate multiple patient derived organoids using immunohistochemical and hormonal validation methods.

Given the difficulty in treating metastatic disease, patients may experience quality of life benefit from catecholamine inhibition previously demonstrated with Sunitinib in the PC-12 pheochromocytoma cell line^41^. We observed variation in norepinephrine levels produced by PTOs from Patient 10 after treatment with tyrosine kinase inhibitors. While Pazopanib-treated PTOs demonstrated reduced norepinephrine levels post-treatment, there were increased levels of catecholamine production with Cabozantinib 1µM and Sunitinib 0.1 µM. Given the clinical side effect of hypertension with tyrosine kinase inhibitor treatment, it could be theorized, that part of the hypertensive findings with Sunitinib are derived through catecholamine release systemically—potentially due to treatment effect. Although this has not been studied with this class of drugs, increased catecholamine levels have been demonstrated after treatment with the radiolabeled somatostatin analog ^177^Lu-DOTATATE^42^. Thus, further studies are aimed at elucidating the mechanism behind transient catecholamine release after treatment with tyrosine kinase inhibitors.

There are a few retrospective single institutional studies informing our knowledge on PCC and PG treatment. The first study to introduce dacarbazine-based therapy for the treatment of metastatic PCC was by Keiser et al. in 1985, where 3 patients demonstrated improvement in blood pressure and two with tumor regression^43^. A large single institution experience from MD Anderson included 52 evaluated patients, with 41 receiving a dacarbazine-based regimen and 33% of patients (17/52) demonstrated decrease in tumor size and improvement in blood pressure^6^. In our study, we observed significant cytotoxicity in 6/13 (46.2%) tumors treated with Vincristine, Dacarbazine, Doxorubicin (VDD) or Vincristine, Dacarbazine, Doxorubicin, and Cyclophosphamide (VDDC). Evidence for additional cytotoxic regimens is limited. There are case reports utilizing Gemcitabine ± Paclitaxel, but the specific regimen exhibited no treatment effect in our PTO cohort^26,27^. Additionally, Temozolomide has demonstrated response in PCC and PG patients harboring SDH B germline PVs^31^ but we did not identify treatment response in 8 PTOs tested. Only tumors 1 and 7 had SDH B PVs; and while tumor 7 demonstrated the greatest post-treatment effect (72.7%), it was not statistically significant. This only highlights the challenges in establishing treatments for rare disease cohorts, given the individual varied responses both clinically and in our PTO study. Additional PTO studies can help identify responders and non-responders in patients with targetable genetic alterations. This point is further emphasized by our PTO cohort’s resistance to immunotherapy, characterized by only one patient’s response to combination therapy with Ipilimumab/Nivolumab. The overall resistance to immunotherapy is in line with current trial data^44^.

As seen with other rare tumors, low incidence of disease makes prospective studies exceedingly difficult to conduct. As an example, Ayala-Ramirez et al. study included 54 patients over a period of 31 years with significant treatment heterogeneity inclusive of 12 treatment protocols^6^. For patients undergoing operative resection, PTOs can be biofabricated and drug results generated within 10 days^23,30,36^. This allows the inclusion of every resected tumor into PTO studies effectively shortening the time horizon to generate disease cohort treatment data while simultaneously identifying patient-specific regimens (i.e. precision oncology). In this way, PTO data could inform and shape clinical treatment pathways at much faster rates—years not decades. Patients with tumors resistant to standard of care therapy can be expedited to appropriate clinical trials based on their tumor’s PTO sensitivity, rather than fragmenting the overall study populations with case reports and underpowered cohort studies utilizing only a few drugs. Additionally, patients with treatment resistant tumors can avoid non-therapeutic drugs and the associated lost time and unnecessary side effects. Over time, PTO studies can generate drug efficacy data for all patients—capturing data capable of informing the design of future clinical trials without altering current standard of care treatment pathways. Ultimately, pairing PTO data with patient factors could help match patients to treatments while balancing side-effect profiles, tolerance, cost and convenience.

The recently published FIRSTMAPPP trial represents a significant achievement for PCCs and PGs^45^. Baudin et al. accrued 78 patients over a 7-year period and demonstrated improvement in median PFS from 3.6 months (Placebo) to 8.9 months (Sunitinib, 37.5 mg daily). Although not statistically significant, the results may still be clinically beneficial. We included Sunitinib in our study based on the preliminary trial results presented at ESMO in 2021^46^ and identified significant treatment responses in 9/14 (64.3%) PTOs treated, which was the best amongst three tyrosine kinase inhibitors we evaluated. Although Cabozantinib has demonstrated response rates in 25% of patients in phase 2 studies, we only observed significant treatments in 2/12 (16.7%) of PTOs. Treatment differences observed amongst similar class drugs may be due to the different pharmacokinetic properties of each drug as well as variations in binding epitopes and affinities. Validating PTO drug screen results through prospective clinical correlation will be an important next step.

Several histologic and clinical data points have informed our understanding of the metastatic potential of PCC and PG to varying degrees, including the pheochromocytoma of the adrenal gland scaled score (PASS), tumor size, and SDH B pathogenic variants^32,47–49^. While there remains equipoise surrounding the prognostic value of PASS^47^PTO viability data could help further define metastatic potential and inform adjuvant therapy decisions. Based on our cohort, PASS ≥4 demonstrated dose-dependent responses to VDD/VDDC and Sunitinib compared to tumors with PASS < 4, where the greatest post-treatment viability reduction was observed only at 10 µM doses. Understanding drug sensitivity to this degree can aid in drug selection based on a patient’s ability to tolerate a particular drug’s side effect profile. Further studies can help elucidate underlying mechanisms that may be responsible for this. Overall, PTOs could help give additionally clarity and improve the utility of existing prognostic data.

There are limitations of this study. First, our study only included 16 patients which makes cohort level interpretations and generalizations difficult even for rare tumors. Although, the study’s correlation with the recent FIRSTMAPPP findings and historical cohorts is promising. Second, treatment effect in this study was based on PTO viability response which is different than measuring RECIST tumor size changes and biochemical response in clinical studies mentioned herein. Additional correlation studies will be necessary to elucidate PTO viability response to clinical, radiographic, and biochemical responses. Additionally, we employed a 3-tiered logarithmic dosing modality with many PTOs achieving statistically significant treatment effect at the 10µM dose, which has not demonstrated to be tolerable in early sunitinib pharmacokinetic studies^28^. Most physiologic levels of sunitinib would fall between our 0.1µM and 1µM doses. Third, our study population only included 1 patient with metastatic pheochromocytoma with most treatment regimens tested being utilized in practice for patients with metastatic disease. However, there are no distinguishing markers to determine whether PCC or PG is metastatic aside from the presence of extra-adrenal disease^50^. Thus, treatment of non-metastatic PCC/PG in our study may potentially inform treatment options for those who develop recurrent disease. Finally, two FDA-approved treatment options in metastatic PCC/PG, belzutifan and I-131 MIBG, were not utilized in this study. Belzutifan was only recently FDA approved at the time of article submission for peer review and thus not able to be tested. I-131 MIBG is not widely available due to lack of commercial suppliers and thus not included in this study^13^.

## Conclusions

Generation of pheochromocytoma and paraganglioma organoids is feasible in the clinical setting and can function as a personalized avenue to mitigate rarity of disease. These organoids maintain cellular characteristics of originating tumors and exhibit decreased catecholamine secretion after treatment. Prospective multi-institutional collection of PCC/PG tumors for living tumor organoid biobanks may offer an avenue for new regimen testing and treatment advances in these rare tumors.

### Conference presentation

Society of Surgical Oncology, 2022 Annual Meeting (Boston, MA) - Poster

## Supplementary Information

Below is the link to the electronic supplementary material.

Supplementary Material 1

## Data Availability

The data generated in this study are available upon request from the corresponding author.
