# Supplementary material for: Biofabrication of pheochromocytoma and paraganglioma tumor organoids and assessment of response to systemic therapy
Source: Sci Rep. 2025 Oct 14;15:35889. doi: 10.1038/s41598-025-19806-w (PMC12521660; doi:10.1038/s41598-025-19806-w)
Supplement: Supplementary file 1 — Supplementary Material 1 [file 41598_2025_19806_MOESM1_ESM.pdf]

Biofabrication of Pheochromocytoma and Paraganglioma Tumor Organoids and Assessment of Response to Systemic Therapy

Richard A. Erali, MD MPH[1,2,6]; Steven D. Forsythe, PhD[1,2,3]; Cecilia R. Schaaf, DVM PhD[1,2,4]; Nicholas Edenhoffer, MS[1,2]; William Meeker, BS[1,2]; Cristian D. Valenzuela, MD[1,2,5]; Wencheng Li, MD[5]; Shay Soker, PhD[1,2,3]; Reese W. Randle, MD[6]; *Konstantinos I. Votanopoulos MD PhD[1,2,6]

Supplementary File

Panel (# of genes)	Genes
Invitae Hereditary Paraganglioma-Pheochromocytoma (14)	MAX, NF1, RET, SDHA, SDHAF2, SDHB, SDHC, SDHD, TMEM127, VHL, EGLN1, FH, KIF1B, MEN1
Invitae Multi-Cancer panel (84)	AIP, ALK, APC, ATM, AXIN2, BAP1, BARD1, BLM, BMPR1A, BRCA1, BRCA2, BRIP1, CASR, CDC73, CDH1, CDK4, CDKN1B, CDKN1C, CDKN2A, CEBPA, CHEK2, CTNNA1, DICER1, DIS3L2, EGFR, EPCAM, FH, FLCN, GATA2, GPC3, GREM1, HOXB13, HRAS, KIT, MAX, MEN1, MET, MITF, MLH1, MSH2, MSH3, MSH6, MUTYH, NBN, NF1, NF2, NTHL1, PALB2, PDGFRA, PHOX2B, PMS2, POLD1, POLE, POT1, PRKAR1A, PTCH1, PTEN, RAD50, RAD51C, RAD51D, RB1, RECQL4, RET, RUNX1, SDHA, SDHAF2, SDHB, SDHC, SDHD, SMAD4, SMARCA4, SMARCB1, SMARCE1, STK11, SUFU, TERC, TERT, TMEM127, TP53, TSC1, TSC2, VHL, WRN, WT1
Invitae Common Hereditary Cancer (48)	APC, ATM, AXIN2, BAP1, BARD1, BMPR1A, BRCA1, BRCA2, BRIP1, CDH1, CDK4, CDKN2A, CHEK2, CTNNA1, DICER1, EPCAM, FH, GREM1, HOXB13, KIT, MBD4, MEN1, MLH1, MSH2, MSH3, MSH6, MUTYH, NF1, NTHL1, PALB2, PDGFRA, PMS2, POLD1, POLE, PTEN, RAD51C, RAD51D, SDHA, SDHB, SDHC, SDHD, SMAD4, SMARCA4, STK11, TP53, TSC1, TSC2, VHL

Supplemental Table 1: Genetic testing panels utilized in the clinical setting.

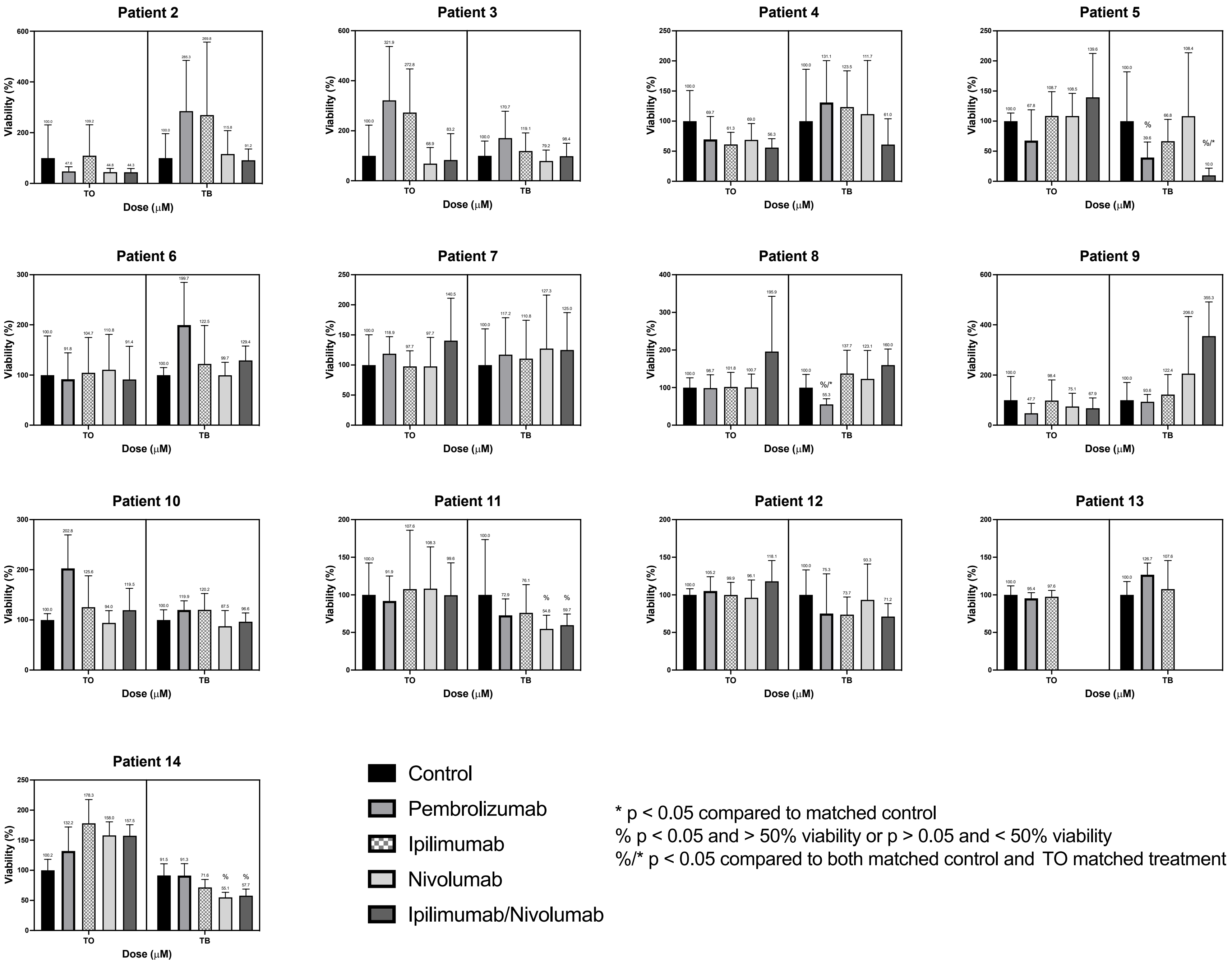

Supplemental Figure 1: ATP viability of PTOs from patients 2-14 treated with immune checkpoint inhibitors Pembrolizumab, Ipilimumab, Nivolumab, and the combination Ipilimumab/Nivolumab. Each patient's PTOs were treated in replicates of at least n=4.
